# Data-driven prediction on critical mechanical properties of engineered cementitious composites based on machine learning

**DOI:** 10.1038/s41598-024-66123-9

**Published:** 2024-07-03

**Authors:** Shuangquan Qing, Chuanxi Li

**Affiliations:** 1 Department of Civil Engineering, Changsha University of Science & Technology, Changsha, 410114 China; 2 State Key Laboratory of Featured Metal Materials and Life-Cycle Safety for Composite Structures, Nanning, 530004 China

**Keywords:** Engineered cementitious composites, Machine learning, Mechanical properties, Random forest, XGBoost, Civil engineering, Mechanical properties

## Abstract

The present study introduces a novel approach utilizing machine learning techniques to predict the crucial mechanical properties of engineered cementitious composites (ECCs), spanning from typical to exceptionally high strength levels. These properties, including compressive strength, flexural strength, tensile strength, and tensile strain capacity, can not only be predicted but also precisely estimated. The investigation encompassed a meticulous compilation and examination of 1532 datasets sourced from pertinent research. Four machine learning algorithms, linear regression (LR), K nearest neighbors (KNN), random forest (RF), and extreme gradient boosting (XGB), were used to establish the prediction model of ECC mechanical properties and determine the optimal model. The optimal model was utilized to employ SHapley Additive exPlanations (SHAP) for scrutinizing feature importance and conducting an in-depth parametric analysis. Subsequently, a comprehensive control strategy was devised for ECC mechanical properties. This strategy can provide actionable guidance for ECC design, equipping engineers and professionals in civil engineering and material science to make informed decisions throughout their design endeavors. The results show that the RF model demonstrated the highest prediction accuracy for compressive strength and flexural strength, with R^2^ values of 0.92 and 0.91 on the test set. The XGB model outperformed in predicting tensile strength and tensile strain capacity, with R^2^ values of 0.87 and 0.80 on the test set, respectively. The prediction of tensile strain capacity was the least accurate. Meanwhile, the MAE of the tensile strain capacity was a mere 0.84%, smaller than the variability (1.77%) of the test results in previous research. Compressive strength and tensile strength demonstrated high sensitivity to variations in both water-cement ratio (W) and water reducer (WR). In contrast, flexural strength exhibited high sensitivity solely to changes in W. Conversely, the sensitivity of tensile strain capacity to input features was moderate and consistent. The mechanical attributes of ECC emerged from the combined effects of multiple positive and negative features. Notably, WR exerted the most significant influence on compressive strength among all features, whereas polyethylene (PE) fiber emerged as the primary driver affecting flexural strength, tensile strength, and tensile strain capacity.

## Introduction

Engineered cementitious composites (ECCs) represent a class of ultra-high-performance cementitious composites that have significantly transformed the landscape of civil engineering. Since its inception by Li and colleagues in 1992, ECC has garnered substantial interest owing to its notable deformability and superior crack control capabilities^1,2^. Numerous experimental studies on mechanical properties of ECC have been conducted worldwide, such as uniaxial compression^3^, uniaxial tension^4^, flexural^5^, shear^6^, fatigue^7^ and impact^8^, as well as a large number of structural or engineering applications^9–15^. The importance of comprehending and forecasting the mechanical characteristics of ECCs cannot be overstressed, as they directly influence the design and functionality of civil engineering structures.

Prior studies have often necessitated numerous experimental trials to attain an optimal ECC mix ratio. This process not only consumes considerable time but also demands substantial financial resources. Li introduced strength and energy criteria as a means to ensure ECC materials achieve a stable state of cracking, thereby mitigating potential errors in testing^16^. Based on numerous test results, empirical ranges for the strength and energy criteria have been established for various types of fibers^16,17^. The double criteria are generally obtained by means of a three-point flexural test and a single-crack tensile test^18,19^. However, the double criterion can only be used to determine whether a given mixture ratio can attain multiple cracks and cannot predict the mechanical properties of the mixture ratio.

Machine learning belongs to a sub-direction of artificial intelligence, including supervised and unsupervised^20^. Machine learning has a wide range of applications in the strength prediction of civil engineering materials. Hu used six machine learning models to predict the compressive strength of FRP-concrete confined column and compared them with prevailing mathematical or empirical models. SHAP analysis was utilized to ascertain feature importance and model sensitivity. The findings indicate that the XGB model exhibited superior robustness and accuracy^21^. At the same time, the problem of small experimental datasets was solved by using an innovative method of data augmentation, facilitating the achievement of a higher prediction accuracy for the punching shear strength of steel fiber-reinforced concrete slabs^22^. Jayasinghe used eight machine-learning models to develop a framework for predicting the shear capacity of recycled aggregate concrete (RAC) beams, and the results revealed that the XGBoost model had the best prediction effect, with the coefficient of determination R^2^ on the test set reaching 0.95 (slender beam) and 0.78 (deep beam)^23^. You utilized 48 datasets and three machine learning models to predict the bond strength between UHPC and deformed reinforcing bars. Among these models, the Adaptive Neuro-Fuzzy Interface System (ANFIS) emerged as the most accurate prediction model. The coefficient of determination R^2^ on the test set reached 0.98^24^. Similarly, Zhang predicted FRP and concrete interfacial bond strength with six machine learning models. Here, the XGBoost model performed best, and the root mean square error was reduced by 73% compared with the original empirical formula^25^. Liu predicted the creep compliance of concrete with four machine-learning models and found that the XGBoost was the optimal prediction model, with the R^2^ on the test set reaching 92%^26^. Mai predicted the compressive strength of fiber-reinforced self-compacting concrete with three machine-learning models. Among the three models, the categorical gradient boosting (CGB) model was the best, and the coefficient of determination was 0.986 for the test dataset^27^. Zhu used an artificial neural network (ANN) and support vector machine (SVM) to predict the 7-day compressive strength of ultrahigh-performance concrete (UHPC). The findings indicate that the ANN model yields superior prediction results compared to the SVM model^28^.

Several scholars have recently used machine learning to predict ECC strength. For instance, Shanmugasundaram used the ANN model to predict the compressive strength of ECC mixed with fly ash and GGBS, respectively. Here, the minimum and maximum differences between the predicted and experimental values were 0.317% and 11.4%, respectively^29^. Mahjoubi employed three machine learning models to predict the compressive strength, tensile strength, and ductility of SHCC. The results showed that XGBoost performed best, and the coefficients of determination on the test set were 0.95, 0.97 and 0.93, respectively^30^. Meng employed an artificial neural network algorithm to forecast both the compressive strength and tensile strength of PVA-ECC. The coefficients of determination on the test set were notably high at 0.98 for compressive strength and 0.99 for tensile strength, indicating significant prediction accuracy. However, the dataset in the described study was relatively small, with compressive strength ranging from 21.3 to 75.2 MPa and tensile strength ranging from 1.56 to 5.81 MPa^31^.

Moreover, the dataset used for machine learning is limited in scope, as it does not encompass high-strength and ultra-high-strength datasets. The algorithms with better results are XGBoost and artificial neural networks. However, artificial neural networks are prone to overfitting, and their training process is intricate. Despite this, there is a noticeable scarcity of predictions for the tensile strain capacity and flexural strength of ECC materials. Existing studies primarily focus on predicting compressive and tensile strength, predominantly within the PVA-ECC domain. The preparation of ECC may use PE fibers, PP fibers, and PVA fibers, which are also divided into oil-film PVA fibers and oil-free PVA fibers. The influence of specimen size on ECC mechanical properties remains unexplored in machine learning literature, indicating a gap in comprehensive understanding within prior research.

Based on a more extensive range of data sets, the aim of the present study was to predict critical mechanical properties of ECC, including compressive strength, flexural strength, tensile strength, and tensile strain capacity. At the same time, the importance of the characteristics affecting the mechanical properties of ECC was explored. Finally, a design strategy for adjusting ECC components was proposed according to the specified mechanical properties.

## Data processing

### Data collection and preprocessing

In the present study, the dataset uniformly sets the cement ratio as 1, while expressing the proportions of other components relative to cement. To use machine learning to predict the mechanical properties of ECC, a total of 1532 sets of data were collected from the existing literature^3,32–68^, including 501 sets of compressive strength, 224 sets of flexural strength, 404 sets of tensile strength, and 403 sets of tensile strain capacity. The experimental group containing CGP was completed by the present author. Owing to space limitations, the experiment details are not provided, but the data can be published, and the relevant experiments are to be published in other articles. There were a total of 13 input features and four output labels. The selection of input features was based on the research conclusions of the literature referenced in the database. The input features were water-cement ratio (W), silica fume (SF), slag (SG), fly ash (FA), coal gangue powder (CGP), silica sand (SS), fiber content (FCO), water reducer (WR), fiber class (FC), fiber aspect ratio (FAR), compressive specimen size (CSS), cement grade (CG), and flexural specimen size (FSS). The output labels were compressive strength (CS: 7.4–166 MPa), flexural strength (FS: 2.6–34.6 MPa), tensile strength (TS: 0.95–18 MPa), and tensile strain capacity (TSC: 0.002% ~ 12.3%). Among them, FC was divided into surface oil-coating polyvinyl alcohol fiber (PVA-O), surface oil-free polyvinyl alcohol fiber (PVA-UO), ultra-high molecular weight polyethylene fiber (PE), fiber-free (NF) (There was a test group without fiber to analyze the effect of fiber), and polypropylene fiber (PP); CG was divided into PO.42.5 and PO.52.5; CSS was divided into 50 mm, 70 mm, and 100 mm; FSS was divided into three cases: four-point flexural plate (PLATE), three-point flexural beam (3-XL), and four-point flexural beam (4-DL). Therefore, 19 input features were included in the present study. The test conditions from which the data set was derived needed to be specified. In the present study, only the datasets under 28-day standard curing conditions were considered, utilizing variable speed mixing methods. However, the input features did not encompass properties of raw materials, such as particle size distribution, particle shape, and chemical composition, which could potentially have influenced the mechanical properties of ECC. Nevertheless, these factors were omitted due to the challenge of quantitatively describing and simplifying them within the machine learning model.

The machine learning data set was derived from the reference literature, and the mix ratios with missing values were excluded when collecting data. Before training the model, the classification features were processed by means of one-hot encoding, and the input features of the non-tree model were normalized using min–max scaling.

### Data analysis

**Figure 1:**
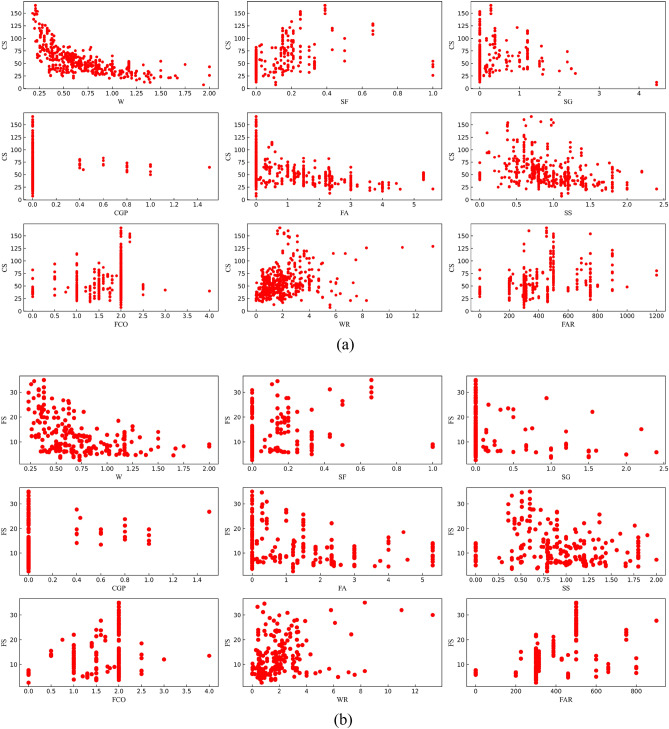

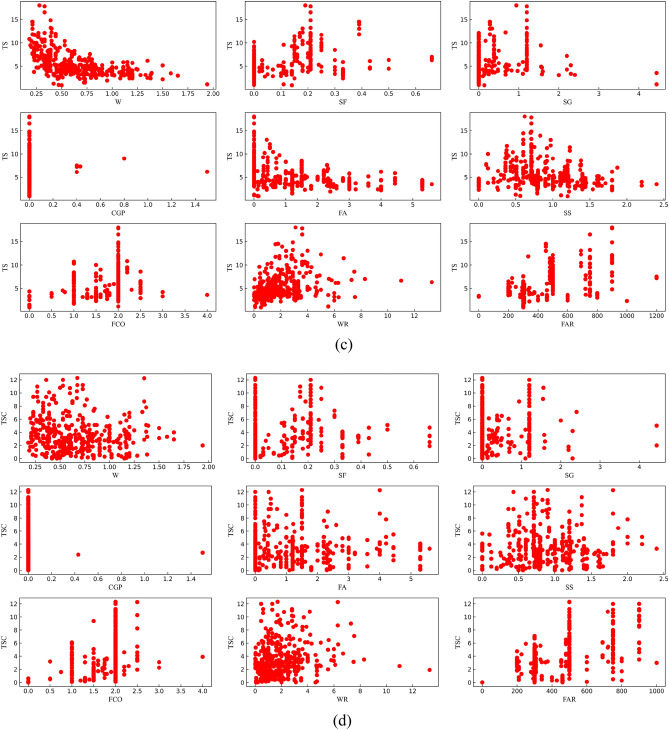
The relationship between features and mechanical properties [(**a**) CS, (**b**) FS, (**c**) TS, (**d**) TSC].

**Figure 2:**
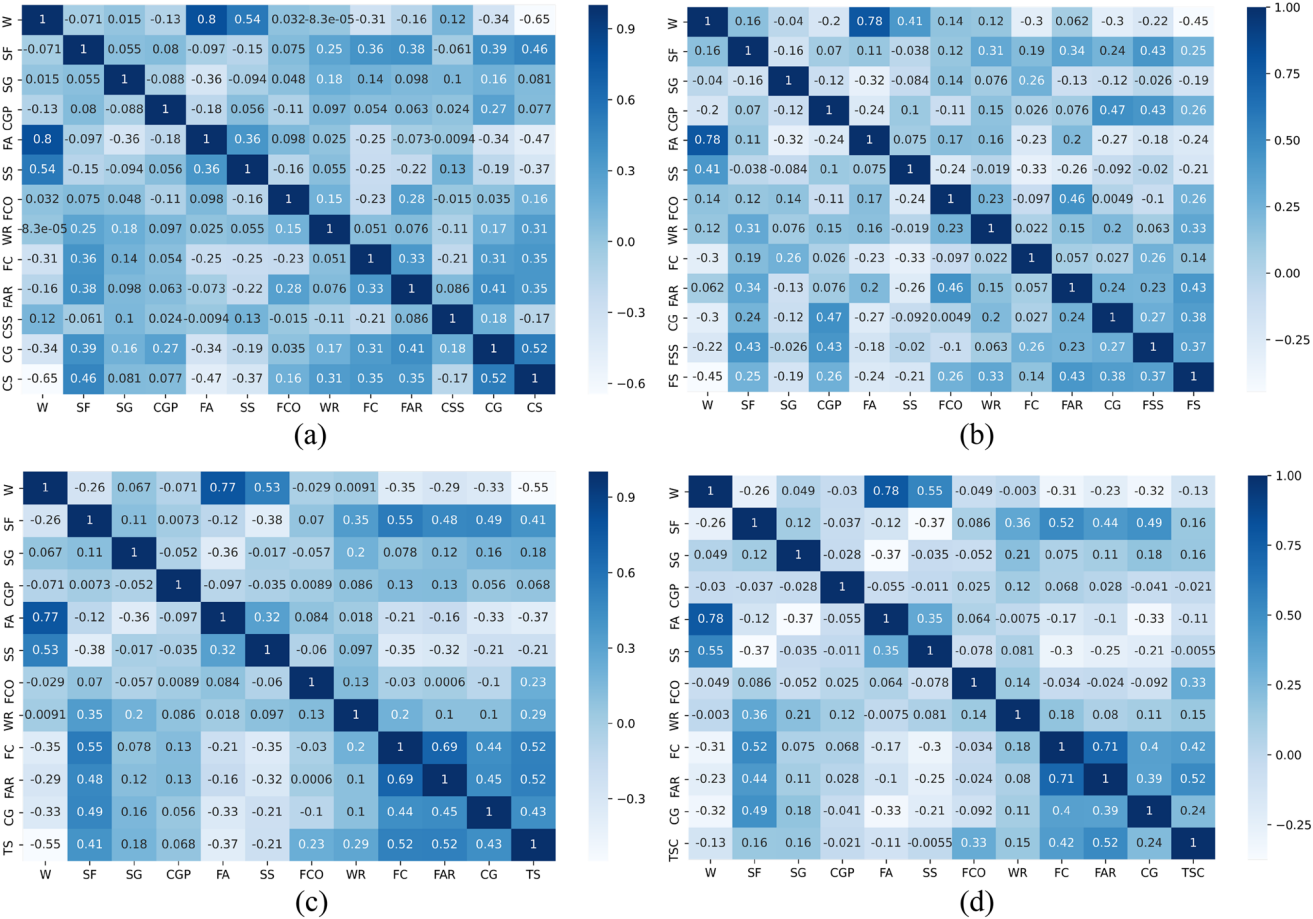
Correlation heat map between all features [(**a**) CS, (**b**) FS, (**c**) TS, (**d**) TSC].

Figure 1 shows the relationship of all continuous characteristic parameters and compressive strength. Figure 2 shows the correlation heat map between all features (including classification features). However, the main focus was primarily on the correlation between each feature and the mechanical properties. Correlations with coefficients between −0.4 and 0.4 were disregarded as they were considered insignificant for the analysis^26^. According to Figs. 1 and 2, there was a negative correlation between (W, FA) and CS and a positive correlation between (CG, SF) and CS; a negative correlation between FAR and FS; a positive correlation between W and FS; a negative correlation between W and TS; and a positive correlation between (FAR, FC, CG, SF) and TS. There was no negative correlation between feature parameters and TSC, but a positive correlation between (FAR, FC) and TSC.

### Methodology

**Figure 3:**
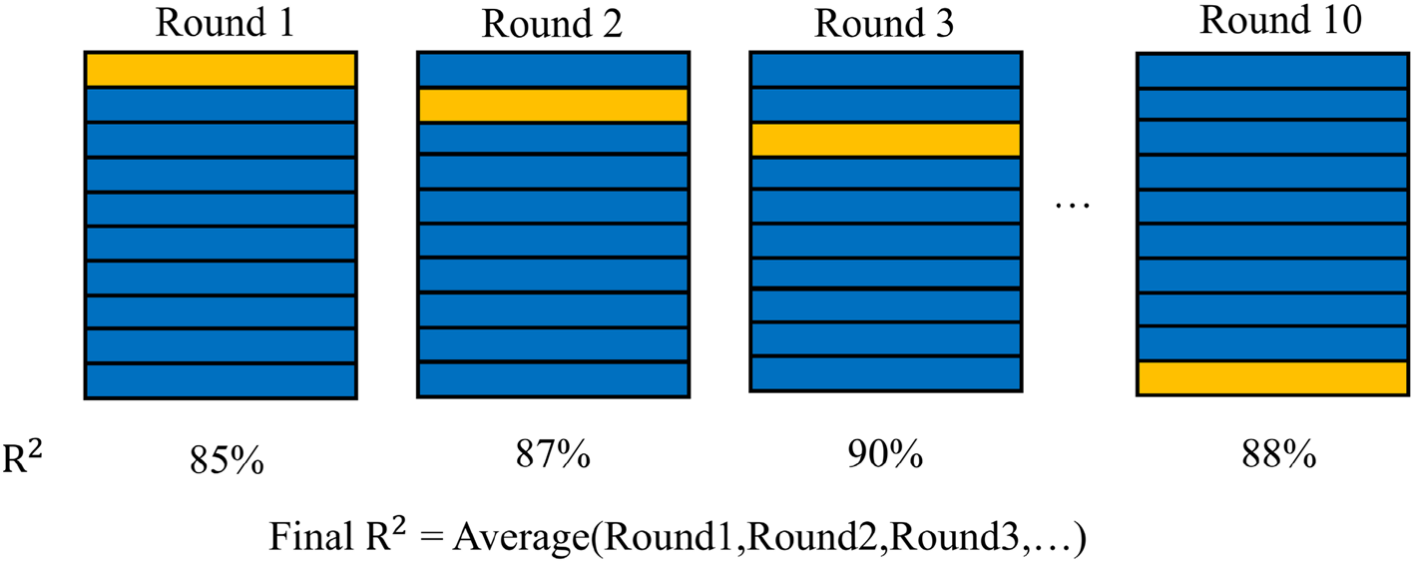
10-fold cross-validation.

**Figure 4:**
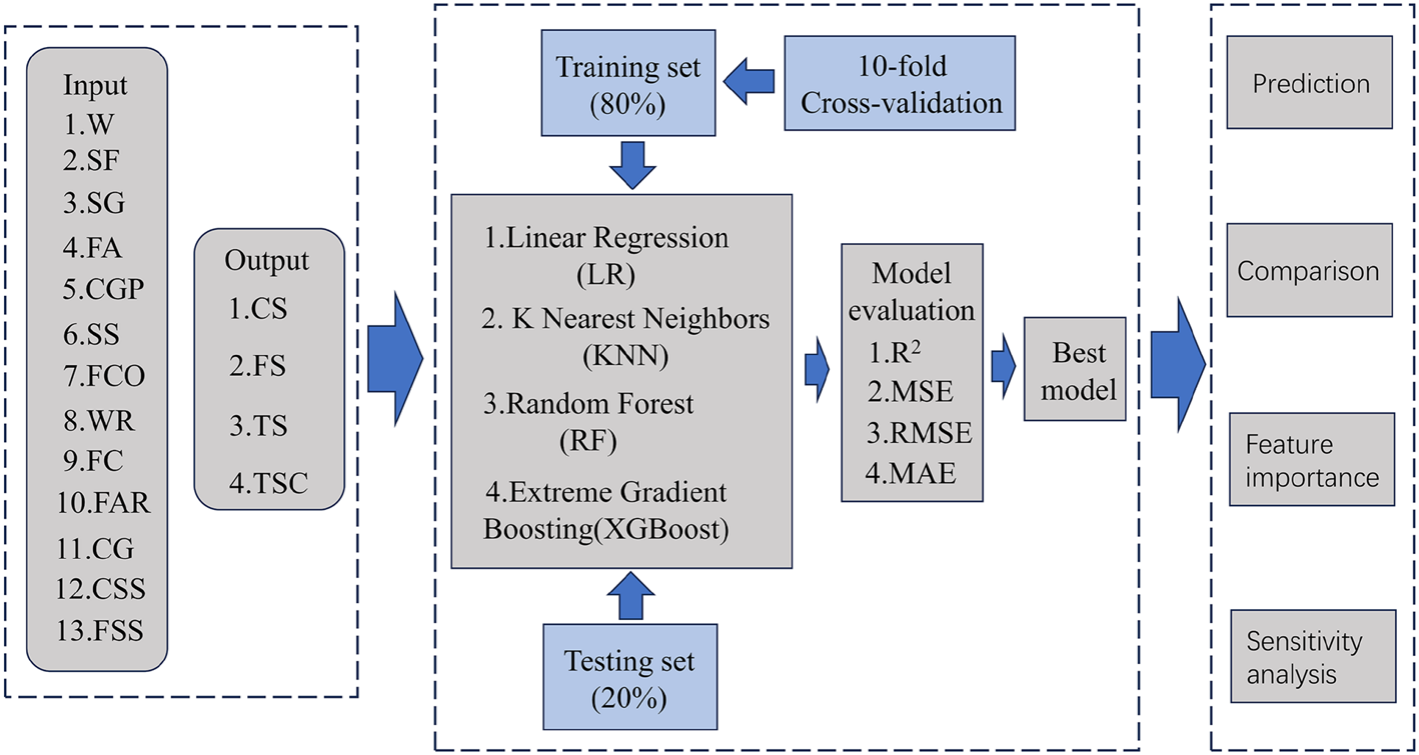
Machine learning technology route for the prediction of ECC mechanical properties.

The technology route of the present study can mainly be divided into three steps: the first step is data collection and division. The training set accounted for 80%, and the test set accounted for 20%. In the second step, the tenfold cross-validation was used to optimize the hyperparameters and reduce overfitting, as shown in Fig. 3. The third step involved predicting the test set and analyzing the importance of features. Figure 4 shows the specific technology route.

## Machine learning

### Machine learning models

Numerous machine learning algorithms are available for predicting the mechanical properties of concrete. In the present study, four representative algorithms were employed to predict the mechanical properties of ECC: linear regression (LR), K nearest neighbors (KNN), random forest (RF), and extreme gradient boosting (XGB).

#### LR model

LR is the simplest and most basic supervised learning algorithm in machine learning. It has good interpretability and is widely used in engineering. The calculating function can be expressed as follows:

*h*_*θ*_(*x*_1_, *x*_2_, …, *x*_*n*_) = *θ*_0_ + *θ*_1_*x* + … + *θ*_*n*_*x*_*n*_, *h*_*θ*_(*x*_1_, *x*_2_, …, *x*_*n*_) is a mechanical indicator, *θ*_0,_ *θ*_1_, ... *θ*_*n*_ are regression parameters, and *x*_1_, *x*_2_, …, *x*_*n*_ are characteristic parameters.

#### KNN model

KNN is also a simple algorithm in machine learning. It calculates the K nearest sample points based on Euclidean distance and derives the final result by averaging these K sample points.

Supposing there are two points P and Q, *P* = {*p*_1_, *p*_2,_ …* p*_*n*_}, *Q* = {*q*_1_, *q*_2,_ …* q*_*n*_}, *n* = 1, 2, 3, …, the Euclidean distance between P and Q is $d = \sqrt {(p_{1} - q_{1} )^{2} + (p_{2} - q_{1} )^{2} + \cdots + (p_{n} - q_{n} )^{2} }$.

#### RF model

RF is an ensemble algorithm belonging to the tree model. A random forest comprises multiple decision trees, and there is no correlation between each decision tree in the forest^69^. The main advantages of random forests lie in their ability to handle high-dimensional data without the need for dimensionality reduction or feature selection. Additionally, they can provide insight into feature importance. However, one disadvantage is that random forest models are generally less powerful for regression problems compared to classification problems.

**Figure 5:**
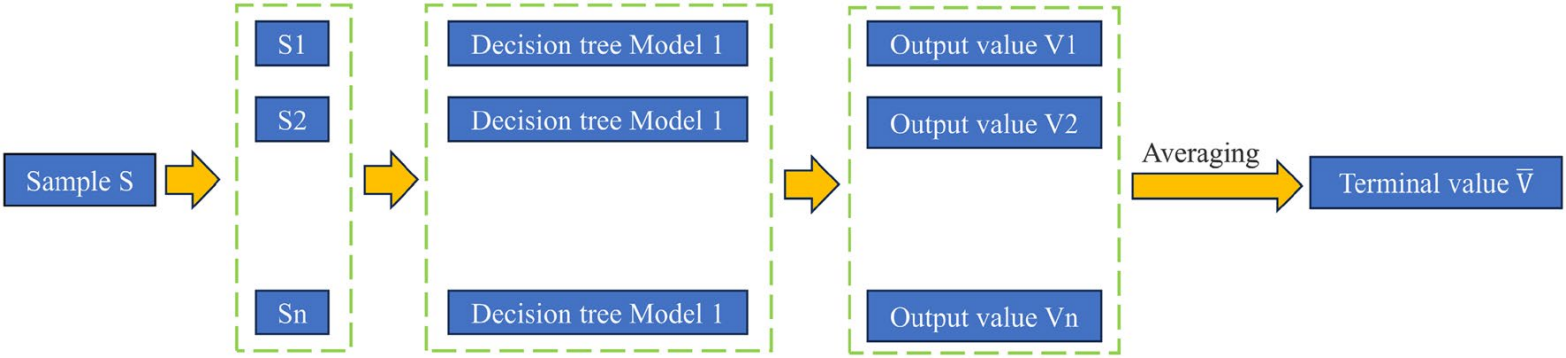
Process of random forest algorithm.

The working mechanism of the RF algorithm is as follows: firstly, M samples (M ≤ N) are randomly drawn from the training set with a size of N for each tree; if a total of K times are randomly selected, then the randomly selected training set is put into K trees for separate training; in the final prediction, the final output of the test set is determined by each decision tree in the forest. For the regression problem, the mean output values of all decision trees are used as the final output. Figure 5 shows the learning flow chart of RF.

#### XGB model

An essential difference between XGB and RF lies in how their decision trees are constructed. In XGB, the decision trees are interrelated, offering certain advantages. The XGB algorithm boasts high accuracy and rapid training speed, and it exhibits robustness to missing values. Nevertheless, it comes with the drawback of high space complexity, necessitating more storage memory^70^.

**Figure 6:**
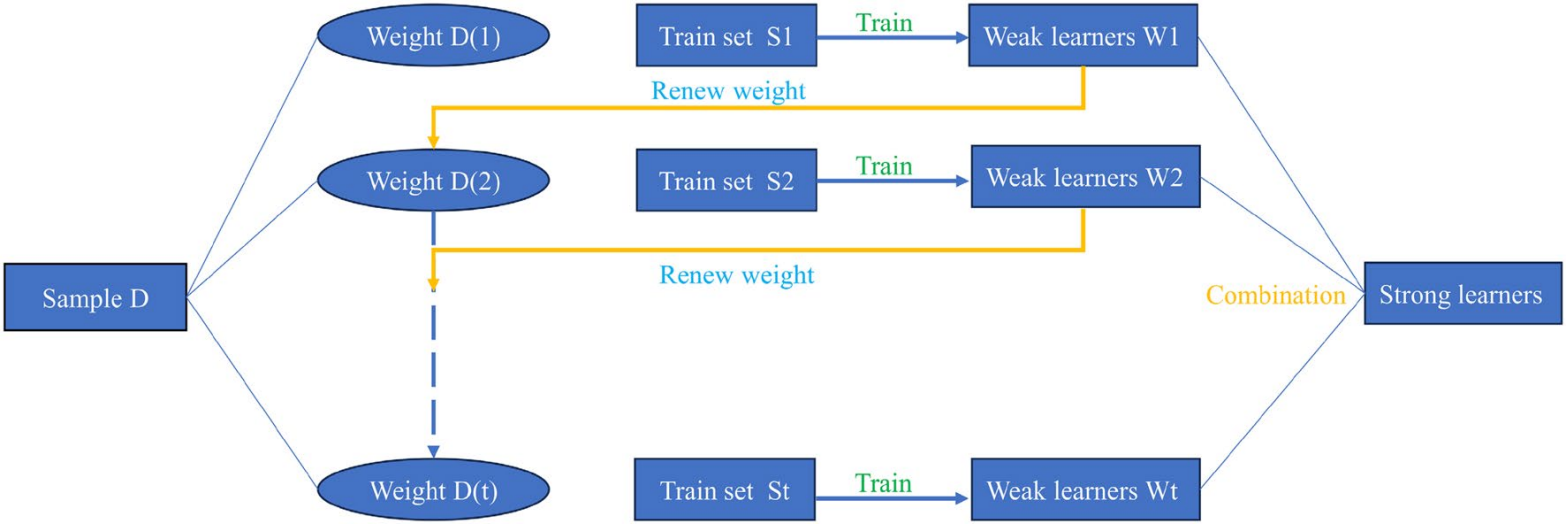
Process of XGB algorithm.

The working mechanism of the XGB algorithm is as follows: firstly, a weak learner W1 is trained on the training set by using the initial weight, and the weight of the training sample is updated according to the residual error left by the weak learner 1. The weight is increased when the residual is large, and the weight is reduced when the residual is small. After updating the weights based on the residuals left by the weak learner 1, the new adjusted weights are used to train the weak learner 2. The described process is repeated until the number of weak learners reaches the preset value. Finally, all weak learners are integrated through a set strategy to obtain the final strong learner. Figure 6 shows the learning flow chart of XGB.

### Evaluation indicators

In the present study, the four evaluation indications most widely used in machine learning were adopted to compare the prediction accuracy of different models: mean square error (MSE), root mean square error (RMSE), mean absolute error (MAE), and coefficient of determination (R^2^). The detailed expressions are shown in Eqs. (1–4), representing the predicted value, actual value, and the average of the actual value.

1 $$ MSE = \frac{1}{m}\sum\limits_{i = 1}^{m} {(y_{i} - \hat{y}_{i} )^{2} } $$

2 $$ RMSE = \sqrt {\frac{1}{m}\sum\limits_{i = 1}^{m} {(y_{i} - \hat{y}_{i} )^{2} } } $$

3 $$ MAE = \frac{1}{m}\sum\limits_{i = 1}^{m} {\left| {(y_{i} - \hat{y}_{i} )} \right|} $$

4 $$ R^{2} = 1 - \frac{{\sum (\hat{y}_{i} - y_{i} )}}{{\sum (\hat{y}_{i} - \overline{y}_{i} )}} $$

## Results and discussion

### Compressive strength

Table 1 lists the optimal parameter values of the model after hyperparameter optimization, and the definition of each parameter is also shown, in which the LR model has no hyperparameter. Table 2 shows the three evaluation indicator values of the four models after hyperparameter optimization on the training and test sets. An observation can be made that the R^2^ values of the LR model on the train and test set were 0.72 and 0.70, respectively, which was the lowest among the four models, while the RMSE and MAE of the LR model on the training and test set were the largest. These indications suggest that the LR model is experiencing underfitting. The R^2^ of the KNN model was significantly higher than that of the LR model, and the RMSE and MAE values were also significantly reduced.

**Table 1:** The optimal parameter values of the model (CS).

Model	Parameter	Definition	Value
KNN	n_neighbors	Number of nearest neighbors	3
RF	n_estimators	Number of decision trees	19
	max_depth	Maximum depth of each decision tree	15
	max_features	Maximum number of features	8
XGB	n_estimators	Number of decision trees	64
	min_child_weight	Minimum sum of instance weight	6
	max_depth	Maximum depth of each decision tree	8
	gamma	Minimum amount of loss required for further branching	0.3

**Table 2:** Three evaluation indicators on the training set and the test set (CS).

Model	Train set			Test set		
	R^2^	RMSE	MAE	R^2^	RMSE	MAE
LR	0.72	14.43	10.55	0.70	15.28	11.24
KNN	0.89	8.98	6.15	0.86	10.43	7.52
RF	0.99	3.23	2.34	0.92	7.90	5.40
XGB	1.00	1.41	1.01	0.89	9.50	6.23

The RF and XGB models were superior to the LR and KNN models. The RF model had the best generalization ability, and its R^2^ was the highest among the four models, at 0.99 and 0.92 on the training and test sets, respectively. The XGB model exhibited signs of overfitting, evident from the R^2^ of 1.00 on the training set and R^2^ of 0.89 on the test set. As such, the XGB model was deemed inappropriate under the division of the compressive strength data set in the present study.

From the R^2^ comparison of the aforementioned models on the training set and the test set, it can be found (Table 2) that the R^2^ values of the LR and KNN models on the two data sets were small. Both models exhibited underfitting in compressive strength prediction. The LR model reached its limit state as it lacked hyperparameters to optimize, and thus was unable to enhance its accuracy in predicting the compressive strength of ECC further. While the KNN model's prediction accuracy for compressive strength may not have matched that of XGB and RF models, it offered simplicity with only one hyperparameter, resulting in faster training speed. Despite this, the KNN model demonstrated acceptable performance on the test set. Overall, in terms of compressive strength prediction accuracy, the RF model emerged as the top performer.

**Figure 7:**
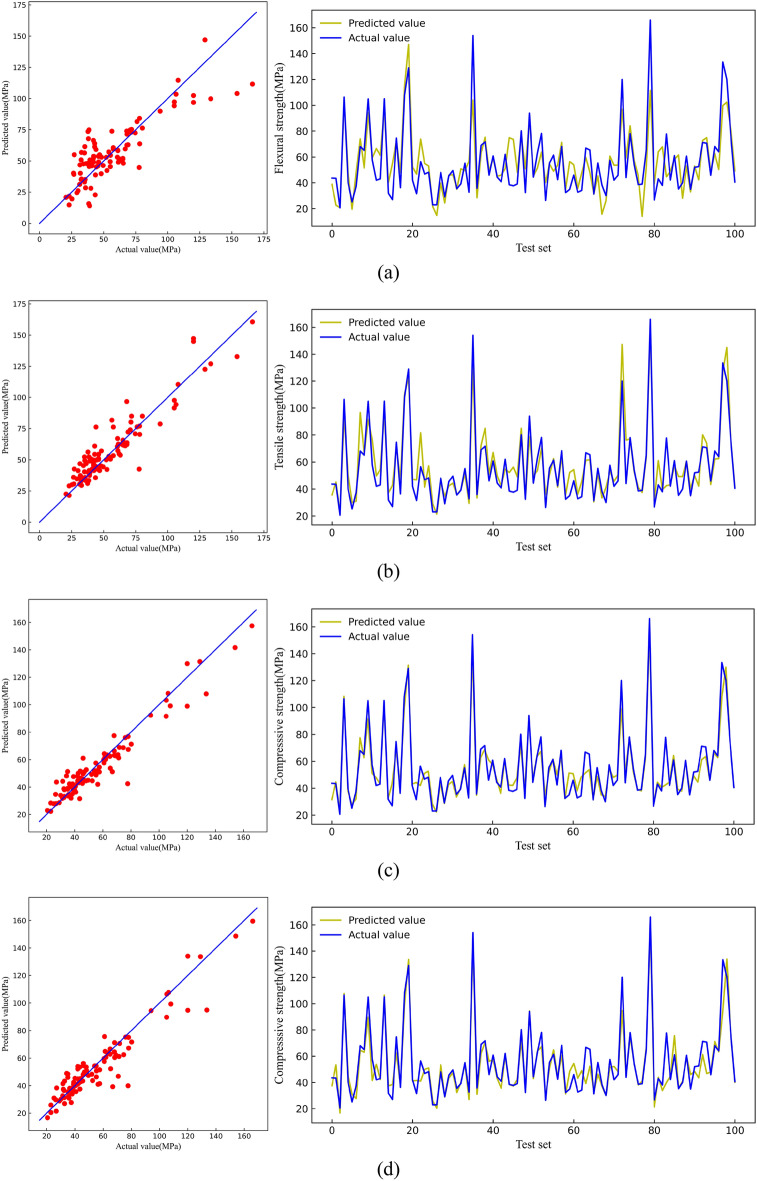
Comparison between predicted value and actual value of CS [(**a**) LR, (**b**) KNN, (**c**) RF, (**d**) XGB].

Figure 7 shows the predicted and actual compressive strength values on the test set. The predicted values of the LR model, KNN model, XGB model, and RF model gradually became more aggregated on both sides of the straight line, indicating that the RF model has the best generalization level on the compressive strength test set.

While ensemble learning methods often exhibit exceptional accuracy, they lack transparency in explaining their output, leading to what is known as the “black box” of machine learning. To address this challenge, the SHapley Additive exPlanations (SHAP) approach was introduced. SHAP is a mathematical framework developed to elucidate the underlying prediction mechanisms of machine learning models. This methodology originated from Shapley game theory and was initially proposed by Lundberg and Lee^71^.

**Figure 8:**
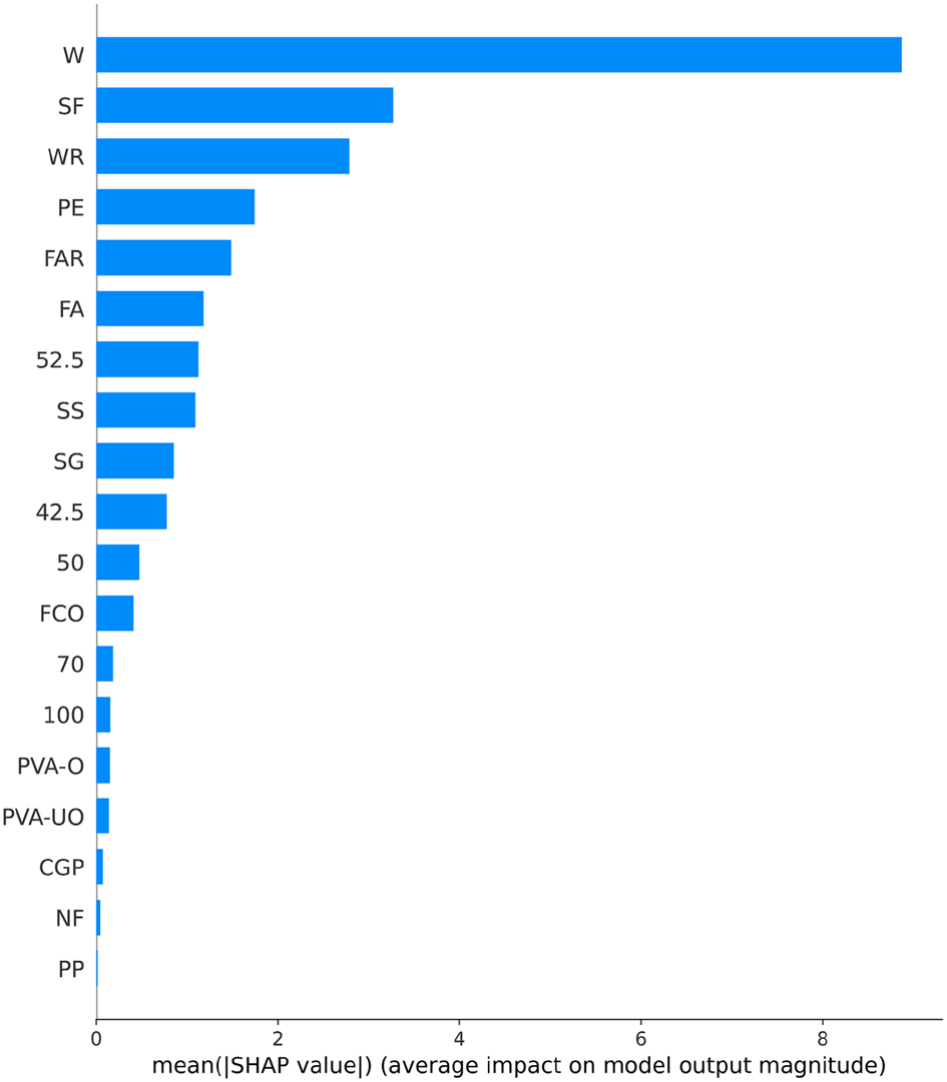
Feature importance of RF modeling.

**Figure 9:**
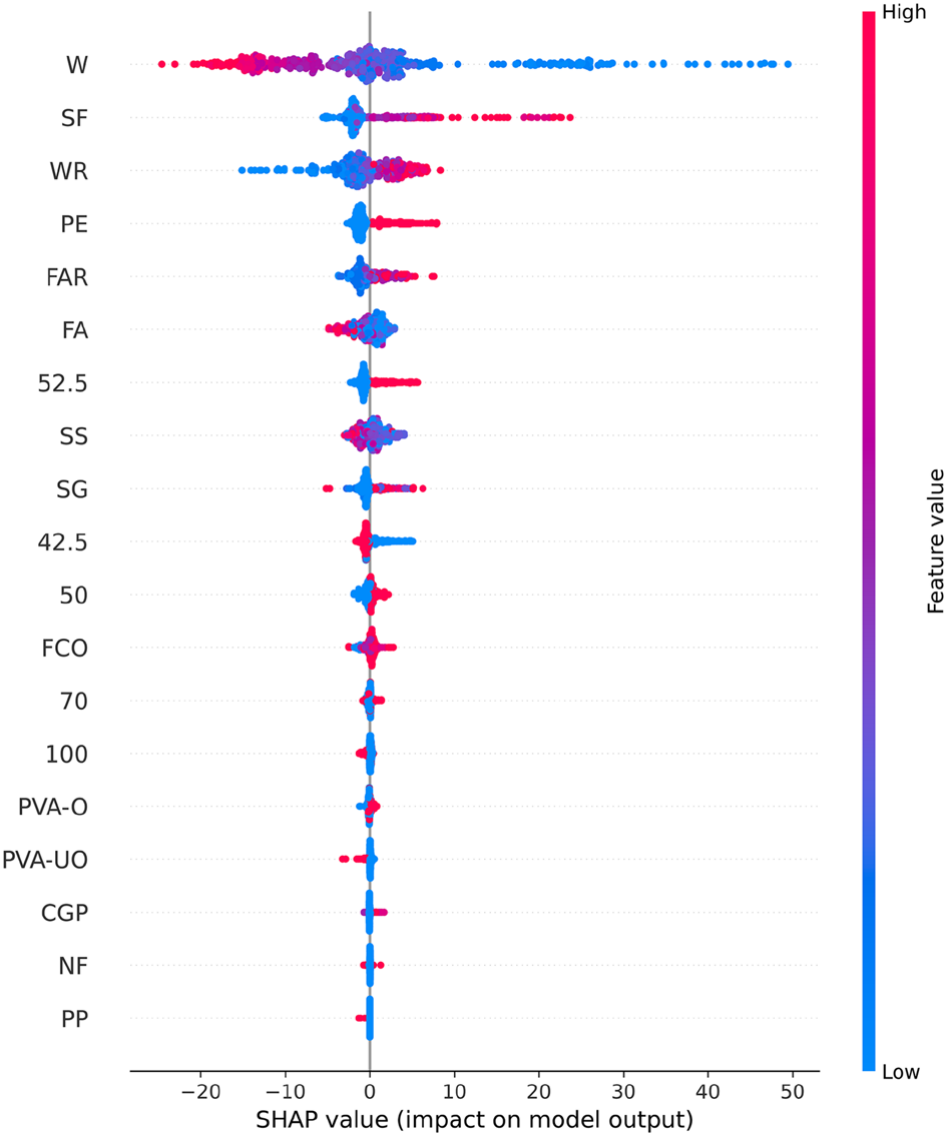
Global SHAP values of RF modeling.

Figure 8 shows the results of the feature importance analysis of the RF model (analyzing the optimal model). The SHAP value of a feature indicates its contribution to the target value. When the SHAP value is closer to 0, it suggests that the feature has a smaller impact on the target value. Conversely, when the SHAP value is farther from 0, it indicates a more significant contribution of the feature to the target value. An observation can be made from Fig. 8 that the water-cement ratio, silica fume, and water reducer contributed the most to the prediction of compressive strength. In contrast, the size of the compressive specimen, PVA fiber, and CGP contributed the least to the prediction of compressive strength. Although the feature importance of Fig. 8 can illustrate the contribution of feature parameters, it cannot indicate whether the feature had a positive or negative impact on the prediction results. However, the Global SHAP value in Fig. 9 can demonstrate how each feature positively or negatively affected the compressive strength. In Fig. 9, each point represents a sample, with red representing a high eigenvalue and blue representing a low eigenvalue. Taking 'W' as an example, the high eigenvalue (red) made a negative contribution to the model output, and the low eigenvalue (blue) made a positive contribution to the model output. A lower eigenvalue (blue) would cause a higher SHAP value, indicating that the water-cement ratio and compressive strength had a negative impact. Contrarily, the high eigenvalue (red) positively contributed to the model output, and the low eigenvalue (blue) negatively contributed to the model output. A higher eigenvalue (red) would cause a higher SHAP value, indicating that silica fume positively affected compressive strength. This pattern applies to other features analyzed similarly.

**Figure 10:**
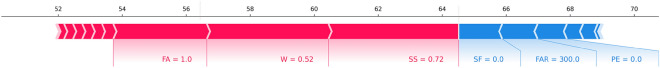
SHAP values of an individual sample.

**Figure 11:**
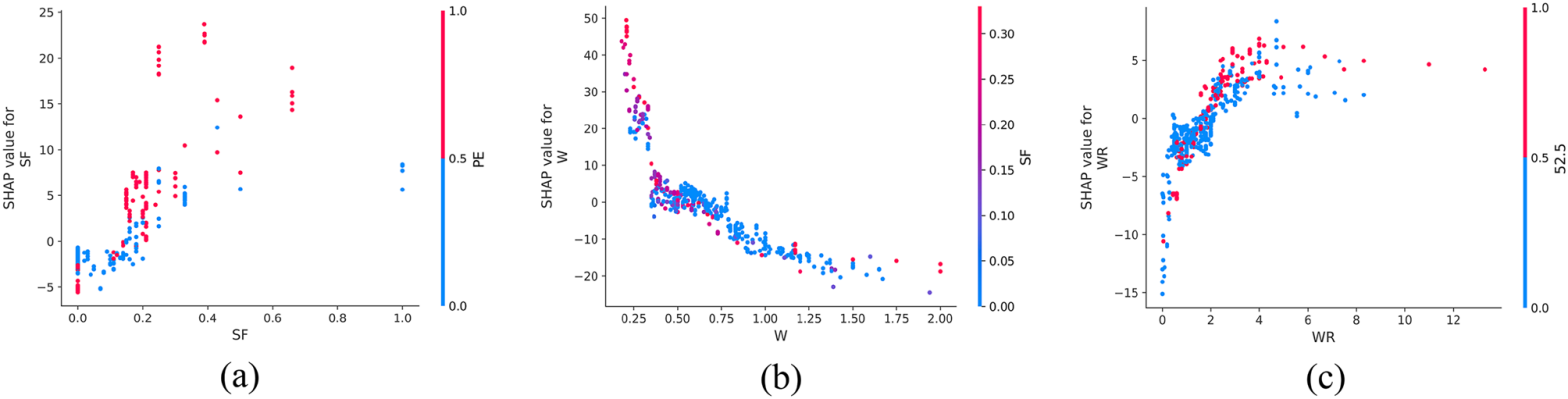
SHAP partial dependency plots for the CS.

SHAP offers the capability to observe both local (individual) interpretability as well as global interpretability, a feature not achieved by traditional variable importance algorithms. In Fig. 10, the SHAP values of a randomly selected single sample from the training model are depicted. The colors, blue and red, represent negative and positive contributions, respectively. Each feature is represented by a bar, with the length indicating its contribution to the prediction result. Red bars signify features that increase the prediction value, while blue bars indicate features that decrease the prediction value. Notably, in this specific example, quartz sand exhibited the longest red bar, indicating its significant contribution to increasing compressive strength, while silica fume had the longest blue bar, suggesting its substantial role in decreasing compressive strength. Additionally, SHAP can illustrate partial dependence plots, which differ from traditional ones by using SHAP values on the y-axis rather than the target value. Figure 11 shows the SHAP partial dependency plots. As shown, the compressive strength was positively correlated with silica fume and water reducing agent and negatively correlated with water cement ratio. In addition, the relationship between silica fume and compressive strength was almost linear. In contrast, the relationship between the water-cement ratio of the water-reducing agent and compressive strength was virtually logarithmic. From Fig. 11b, it can be observed from Fig. 11b that when W < 0.5, the SHAP value decreased with the increase in W, and the compressive strength was highly sensitive to the change of W. When W > 0.5, although W was still inversely correlated with compressive strength, the sensitivity was significantly weaker than W < 0.5. According to Fig. 11c, it can be found that when WR < 0.1, the SHAP value increased with the increase in W, and the compressive strength was highly sensitive to the change of WR; when WR > 0.1, the sensitivity of compressive strength to WR was significantly weakened.

### Flexural strength

Table 3 shows the optimal parameter values of the model after hyperparameter optimization. Table 4 shows the evaluation indicators of the four models on the training and test sets. An observation can be made that the R^2^ values of the LR model and KNN model on the training set were smaller than those on the test set, which suggests the presence of data leakage, potentially leading to overly optimistic results^72^. The RF and XGB models performed better, with R^2^ of 0.97 on the training set and R^2^ of 0.91 on the test set. The RMSE and MAE of the RF model on the training set were 1.16 and 0.80, and the RMSE and MAE on the test set were 2.32 and 1.62, smaller than the XGB.

**Table 3:** The optimal parameter values of the model(FS).

Model	Parameter	Definition	Value
KNN	n_neighbors	Number of nearest neighbors	6
RF	n_estimators	Number of decision trees	229
	max_depth	Maximum depth of each decision tree	18
	max_features	Maximum number of features	6
XGB	n_estimators	Number of decision trees	13
	min_child_weight	Minimum sum of instance weight	4
	max_depth	Maximum depth of each decision tree	9
	reg_alpha	Penalty coefficient of L1 Regularization	0.5

**Table 4:** Three evaluation indicators on the training set and the test set (FS).

Model	Train set			Test set		
	R^2^	RMSE	MAE	R^2^	RMSE	MAE
LR	0.82	3.12	2.27	0.92	2.21	1.73
KNN	0.85	2.83	2.00	0.90	2.48	1.82
RF	0.97	1.16	0.80	0.91	2.32	1.62
XGBoost	0.97	1.33	0.87	0.91	2.32	1.73

**Figure 12:**
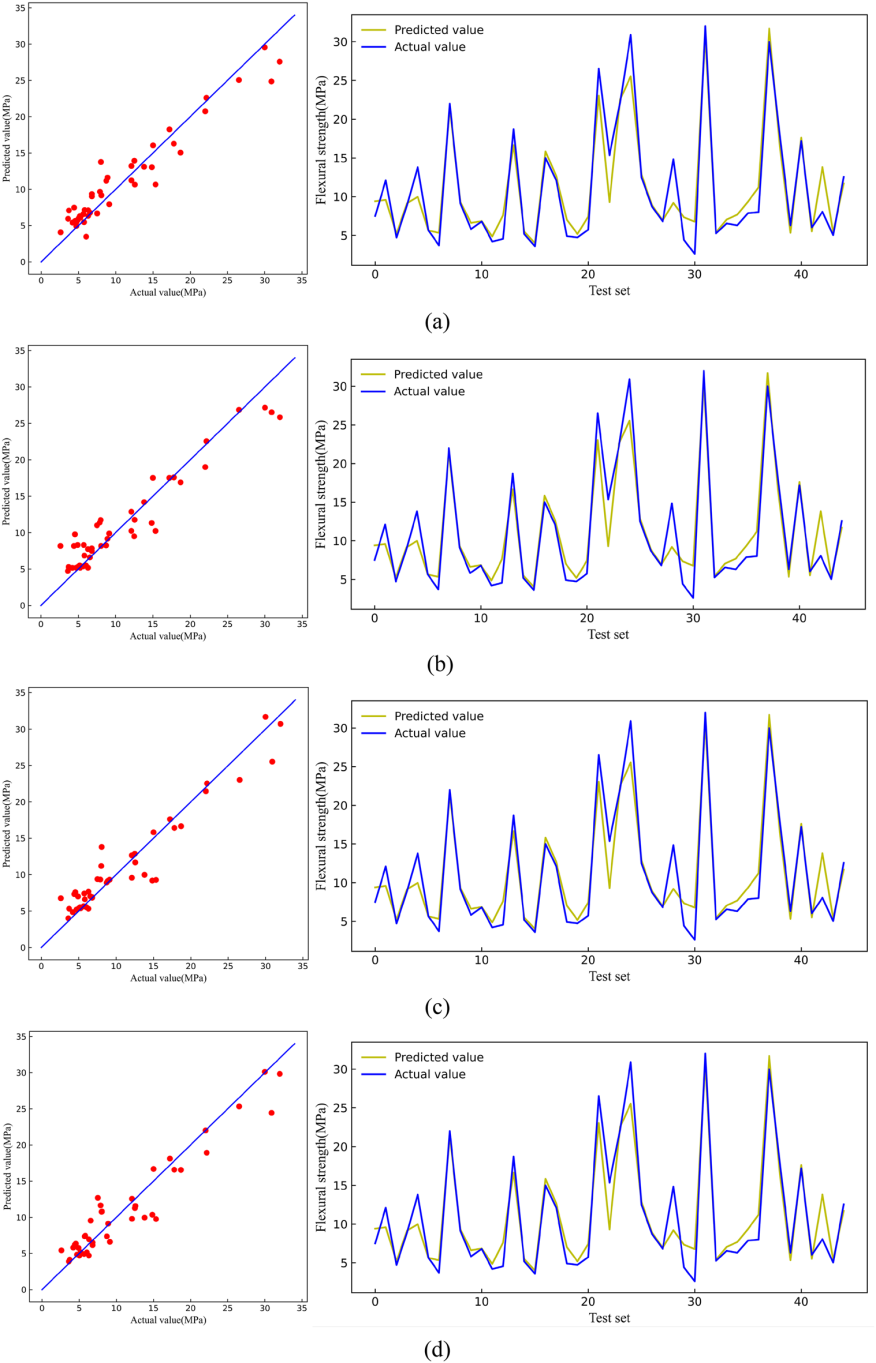
Comparison between predicted value and actual value of FS [(**a**) LR, (**b**) KNN, (**c**) RF, (**d**) XGB].

Figure 12 shows the predicted and actual flexural strength values on the test set. The four models had good prediction results on the test set, but the LR and KNN models exhibited data leakage phenomena that could not be used to make accurate predictions. Thus, only the generalization ability of the RF and XGB models on the test set was deemed practical, and the RF model was the most superior in terms of predicting flexural strength.

In summary, under the premise of the present study’s flexural strength data set, the LR and KNN models exhibited data leakage, and thus were not feasible for ECC flexural strength prediction. The XGB and RF models demonstrated exceptional prediction accuracy for the flexural strength of ECC, effectively addressing a gap in the literature where previous studies did not predict the flexural strength of ECC.

**Figure 13:**
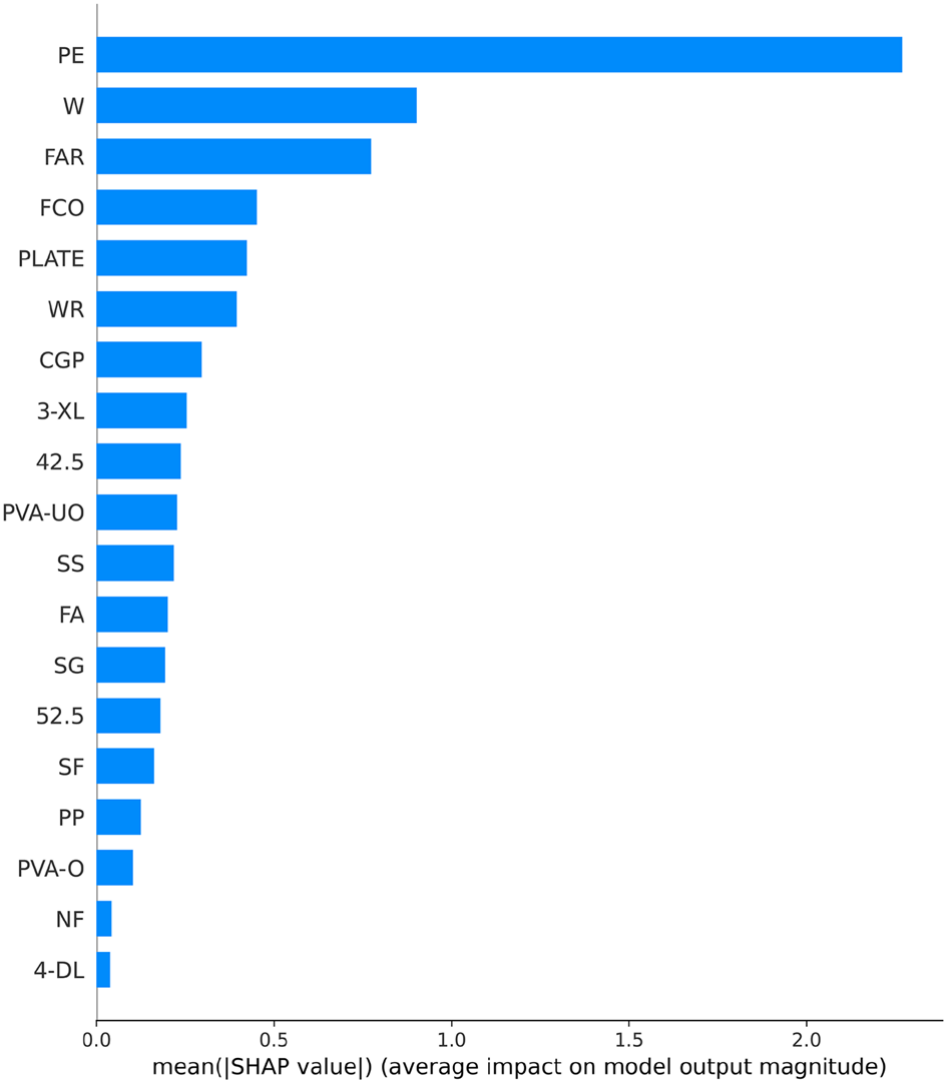
Feature importance of RF modeling.

Figure 13 shows the importance of analyzing RF model characteristics of flexural strength. It indicates that PE fiber, water-cement ratio, and fiber aspect ratio contributed the most to the prediction of flexural strength. In contrast, other fiber types contributed the least to the prediction of compressive strength.

**Figure 14:**
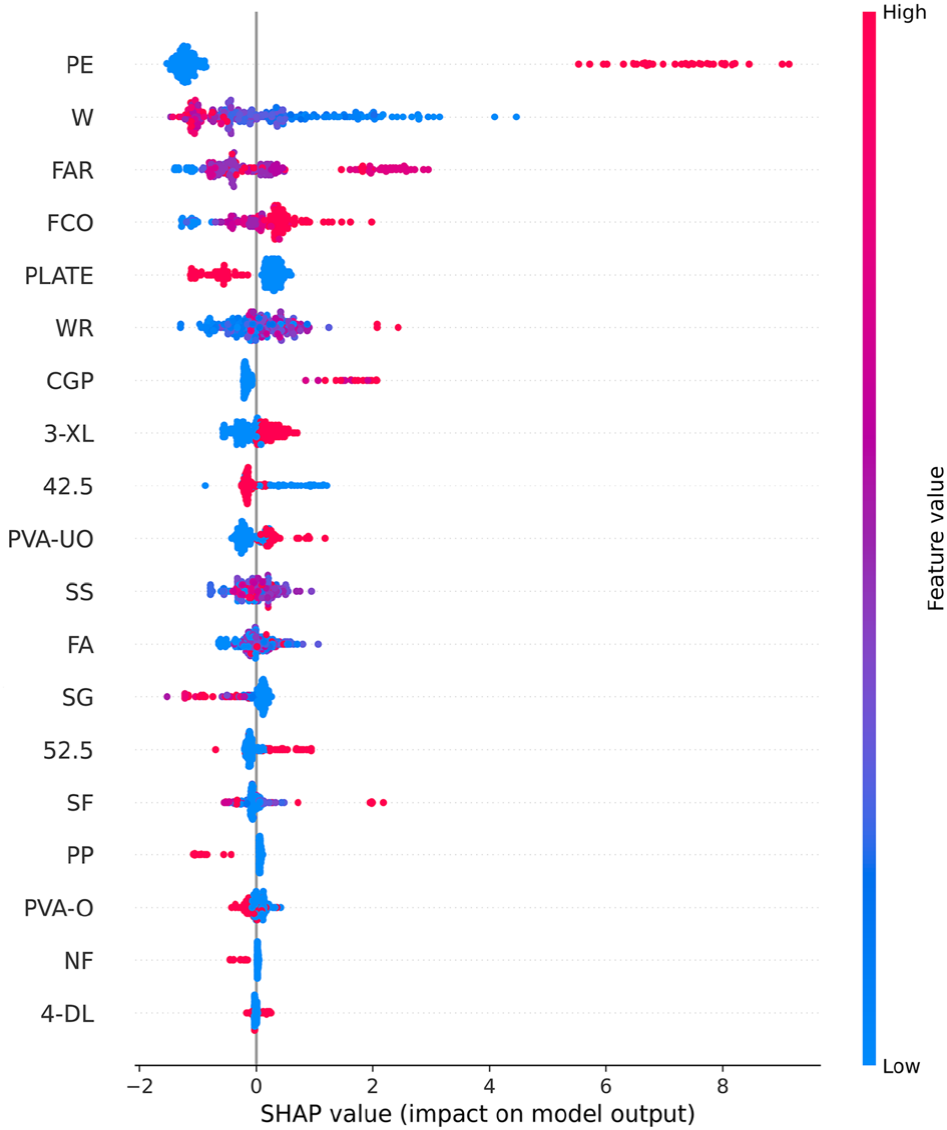
Global SHAP values of RF modeling.

**Figure 15:**
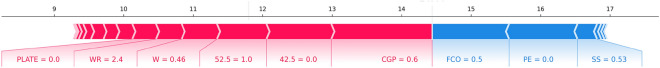
SHAP values of an individual sample.

Figure 14 shows that PE fiber positively affected flexural strength, while the water-cement ratio had a negative effect. Other characteristics can also be analyzed according to the Global SHAP value. Figure 15 shows the SHAP values of a random single sample of the training model. The most extensive red bar was coal gangue powder, which would increase the flexural strength for the specific sample of coal gangue powder. The most extensive blue bar was fiber content, which would reduce the flexural strength in this particular sample of fiber content.

**Figure 16:**
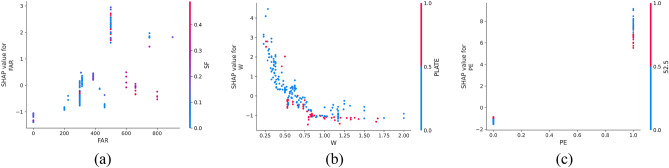
SHAP partial dependency plots for the FS.

In Fig. 16, the SHAP dependency partial plots reveal several key observations regarding the relationship between input features and flexural strength. Notably, flexural strength exhibited a positive correlation with fiber aspect ratio and the presence of PE fiber, while showing a negative correlation with the water-cement ratio. Moreover, the relationship between fiber aspect ratio and flexural strength appeared to be roughly linear, indicating that flexural strength increased with higher fiber aspect ratios. Conversely, the relationship between water-cement ratio and flexural strength appeared to be almost logarithmic. It can be found from Fig. 16b that when W < 0.75, the SHAP value decreased rapidly with the increase in W, indicating that the flexural strength was highly sensitive to the change of W in this range. When W > 0.75, although W was still inversely correlated with flexural strength, the sensitivity had almost disappeared.

### Tensile strength

Table 5 lists the optimal parameter values of the model after hyperparameter optimization. Table 6 shows the evaluation indicator values of the four models on the training and test sets. The R^2^ values of the LR model on the training and test sets were only 0.71 and 0.63, which shows that the LR model could not predict the tensile strength well. The R^2^ values of the KNN model on the training and test sets were 0.89 and 0.75, which indicates overfitting. The R^2^ values of the RF model on the training and test sets were 0.96 and 0.84; although RF exhibited slight overfitting, the prediction accuracy on the test set was relatively good. The R^2^ values of the XGB model on the training and test sets were 0.97 and 0.87. R^2^ was the largest among the four models, and the RMSE and MAE on the test set were only 1.04 and 0.68, the smallest among the four models.

**Table 5:** The optimal parameter values of the model(TS).

Model	Parameter	Definition	Value
KNN	n_neighbors	Number of nearest neighbors	2
RF	n_estimators	Number of decision trees	9
	max_depth	Maximum depth of each decision tree	15
XGB	n_estimators	Number of decision trees	37
	min_child_weight	Minimum sum of instance weight	2
	max_depth	Maximum depth of each decision tree	5
	gamma	Minimum amount of loss required for further branching	0.3
	reg_alpha	Penalty coefficient of L1 regularization	0.05

**Table 6:** Three evaluation indicators on the training set and the test set (TS).

Model	Train set			Test set		
	R^2^	RMSE	MAE	R^2^	RMSE	MAE
LR	0.71	1.46	1.06	0.63	1.73	1.25
KNN	0.89	0.89	0.60	0.75	1.41	0.91
RF	0.96	0.56	0.38	0.84	1.13	0.74
XGB	0.97	0.44	0.32	0.87	1.04	0.68

From the collected data sets, it is evident that the variability of tensile strength in ECC surpassed that of compressive strength. In Table 6, it is apparent that both LR and KNN models exhibited underfitting. In the dataset used in the present study, the LR and KNN models failed to accurately predict the tensile strength of ECC. This could be attributed to the models' simplicity and the considerable variability in tensile strength test values. As such, machine learning struggled to fully capture the underlying relationships within the data, necessitating improvements in model complexity. This is why RF and XGB models achieved more accurate predictions. Despite their increased complexity, tree models yielded superior prediction results.

**Figure 17:**
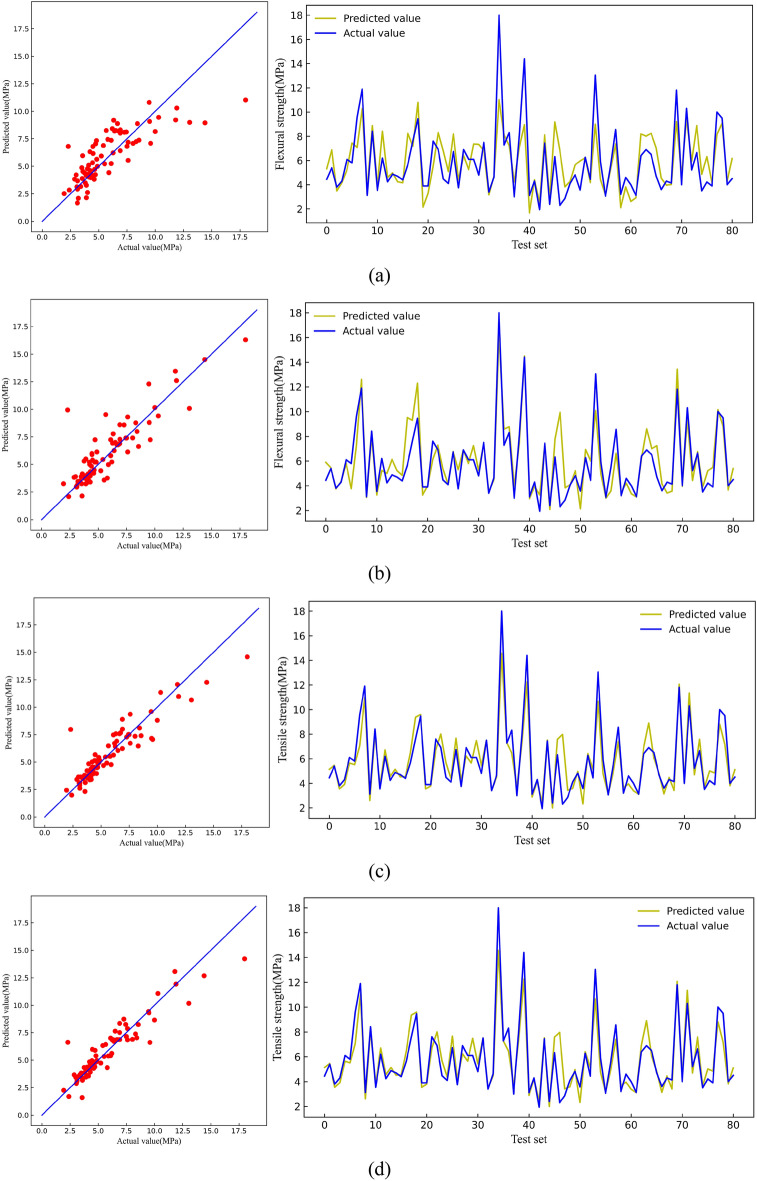
Comparison between predicted value and actual value of TS [(**a**) LR, (**b**) KNN, (**c**) RF, (**d**) XGB].

Figure 17 shows the predicted and actual tensile strength values on the test set. The RF and XGB data were considerably aggregated near the line, indicating exceptional generalization ability of the two models for tensile strength. Comparatively, the XGB model was superior.

In the dataset used in the present study, the LR and KNN models failed to predict the tensile strength of ECC accurately. This could be attributed to the models' simplicity and the considerable variability in tensile strength test values. As such, machine learning struggled to fully capture the underlying relationships within the data, necessitating improvements in model complexity. This is why RF and XGB models achieved more accurate predictions. Despite their increased complexity, tree models yielded superior prediction results.

**Figure 18:**
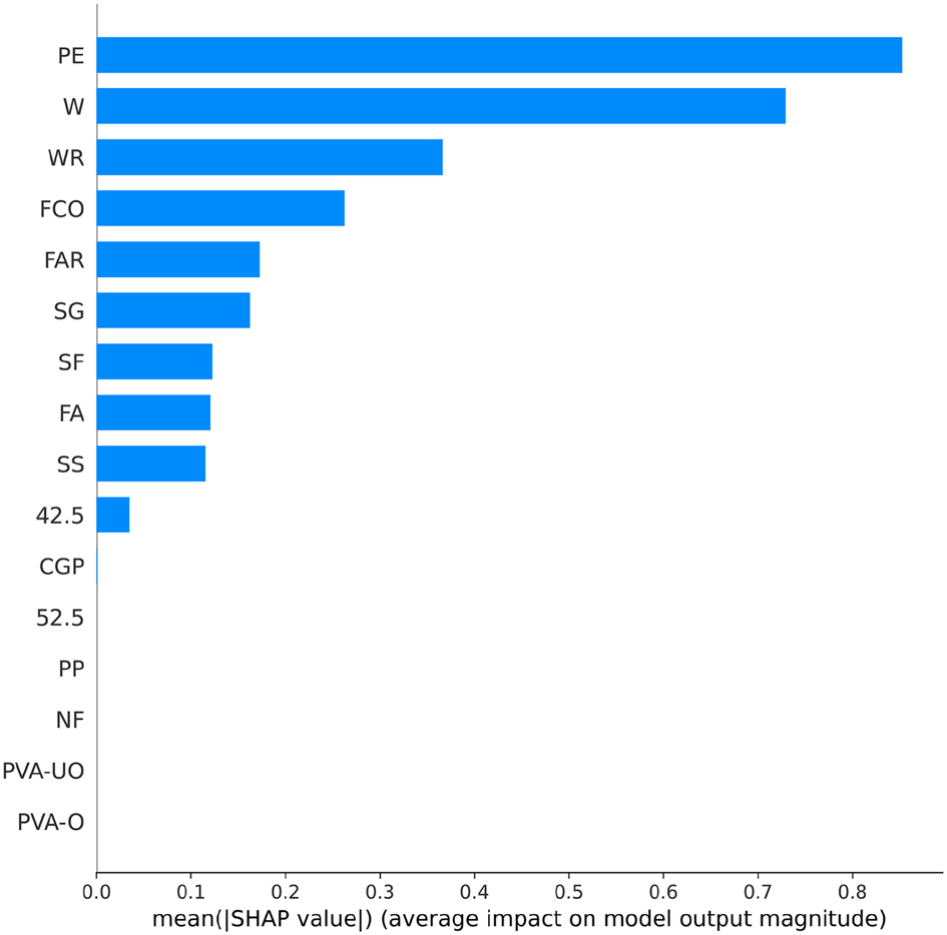
Feature importance of XGB modeling.

Figure 18 shows analysis of the XGB model's feature importance for tensile strength. PE fiber, water-cement ratio, and water reducer contributed the most to predicting tensile strength, while other fibers contributed minimally.

**Figure 19:**
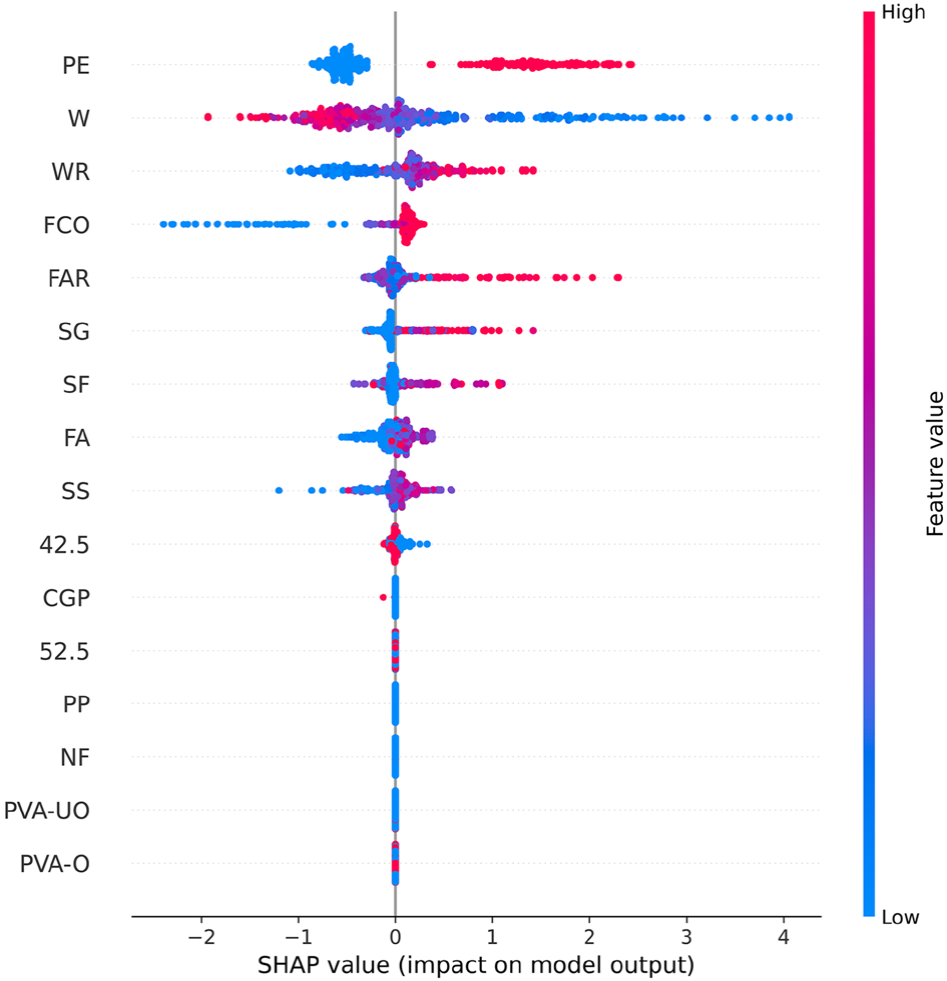
Global SHAP values of XGB modeling.

**Figure 20:**
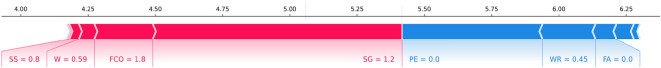
SHAP values of the individual sample.

Figure 19 shows that PE fiber positively affected tensile strength, while the water-cement ratio had a negative effect. Other characteristics could also be analyzed according to the Global SHAP value. Figure 20 shows the SHAP value of a random single sample of the training model. The most extensive red bar was slag. For this specific sample, the slag would increase the tensile strength. The most extensive blue bar was PE fiber. In this particular sample, PE fiber would reduce the tensile strength.

**Figure 21:**
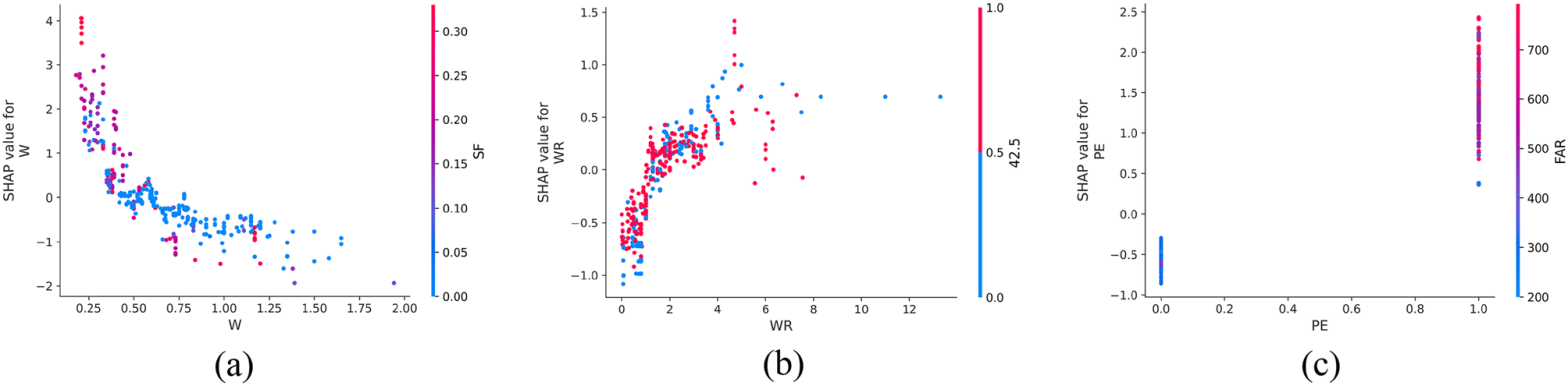
SHAP partial dependency plots for the TS.

Figure 21 shows the SHAP dependency partial plots. An observation can be made that the tensile strength was positively correlated with the water-reducing agent and whether PE fiber was present or negatively correlated with the water-cement ratio. In addition, the water-cement ratio and water-reducing agent were almost logarithmically related to the tensile strength. Figure 21a shows that when W < 0.5, the SHAP value decreased rapidly with the increase in W, indicating that the tensile strength was highly sensitive to the change of W in this range. When W > 0.5, although W was still inversely related to the tensile strength, the sensitivity became considerably weak. An observation can be made from Fig. 21b that when WR < 2, the SHAP value increased rapidly with the increase in WR, indicating that the tensile strength was highly sensitive to the change of WR in this range. When WR > 2, the sensitivity of tensile strength to WR changes in this range became significantly weaker.

### Tensile strain capacity

Table 7 lists the optimal parameter value of the model after hyperparameter optimization. Table 8 shows the evaluation indicator values of the four models on the training and test sets. The LR model was the weakest since the R^2^ values were only 0.63 and 0.61 on the training and test sets. The R^2^ values for the KNN model were 0.85 and 0.70 on the training and test sets, respectively, indicating a slight degree of overfitting. However, the model's generalization ability on the test set remained moderate. The R^2^ of the RF model reached 0.92 on the test set, but was only 0.72 on the test set, indicating overfitting of the model. The XGB model was the best, although slight overfitting existed. The R^2^ values of the XGB model were 0.95 and 0.80 on the training and test set. As shown in previous research^73^, the variation range of tensile strain capacity in the test was [± 0.56%, ± 1.77%]. However, the MAE on the test set of the XGB model in the present study was only 0.84%, which is entirely within a reasonable error range.

**Table 7:** The optimal parameter values of the model.

Model	Parameter	Definition	Value
KNN	n_neighbors	Number of nearest neighbors	3
RF	n_estimators	Number of decision trees	13
	max_depth	Maximum depth of each decision tree	9
	max_features	Maximum number of features	6
XGBoost	n_estimators	Number of decision trees	24
	min_child_weight	Minimum sum of instance weight	4
	gamma	Minimum amount of loss required for further branching	0.3

**Table 8:** Three evaluation indicators on the training set and the test set.

Model	Train set			Test set		
	R^2^	RMSE	MAE	R^2^	RMSE	MAE
LR	0.63	1.62	1.24	0.61	1.54	1.16
KNN	0.85	1.05	0.75	0.70	1.34	1.03
RF	0.92	0.76	0.56	0.72	1.28	0.94
XGBoost	0.95	0.57	0.42	0.80	1.10	0.84

**Figure 22:**
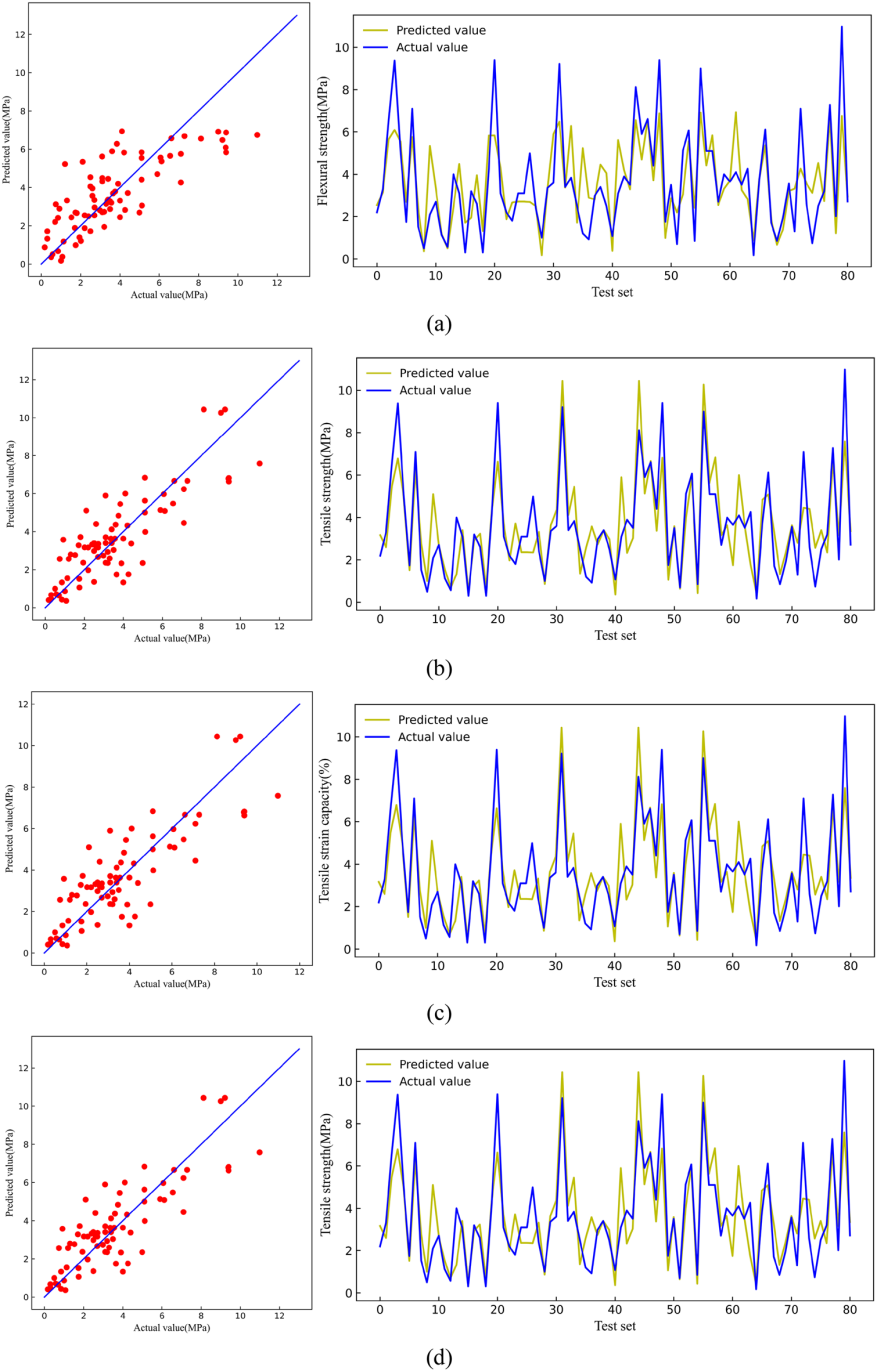
Comparison between predicted value and actual value of TSC [(**a**) LR, (**b**) KNN, (**c**) RF, (**d**) XGB].

Figure 22 shows the predicted values and the actual values of tensile strain capacity on the test set. Among them, the XGB model prediction data was relatively more aggregated near the line and had the best effect on the prediction of tensile strain capacity. Notably, the prediction of tensile strain capacity could not be better than the other three mechanical indicators. This discrepancy can be attributed to the close relationship between tensile strain capacity and the uniform defects within the matrix of ECC. Many test groups do not introduce uniform artificial defects, leading to significant variability in tensile strain capacity test values^74–76^.

**Figure 23:**
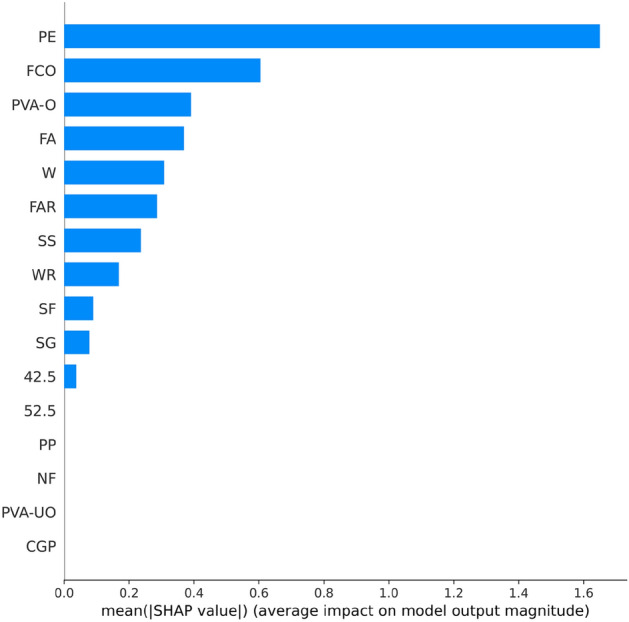
Feature importance of XGB modeling.

**Figure 24:**
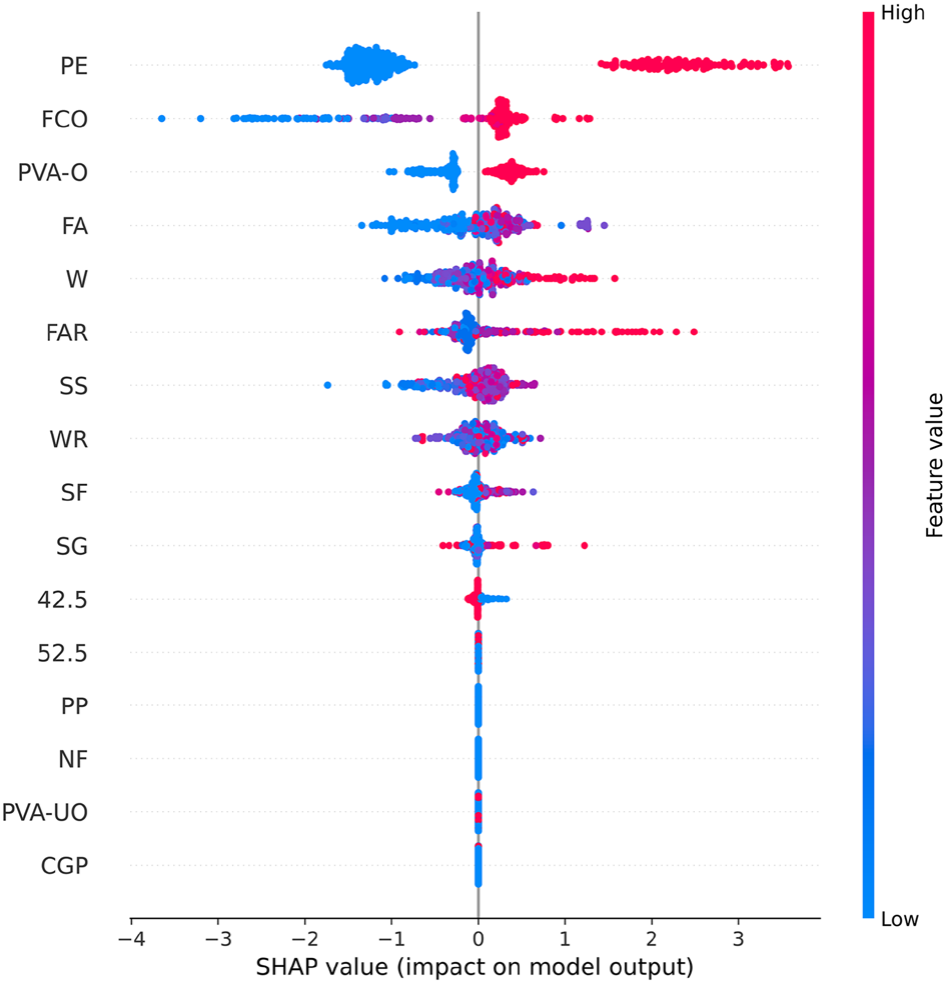
Global SHAP value of XGB modeling.

In Fig. 23, the analysis of the importance of features for the XGB model reveals that PE fiber, fiber content, and oil film PVA fiber made the most significant contributions to predicting tensile strain capacity, while other fibers contributed minimally. Figure 24 shows that PE fiber, fiber content, and oil film PVA fiber positively impacted tensile stress capacity. Additionally, other characteristics can be further analyzed based on Global SHAP values.

**Figure 25:**
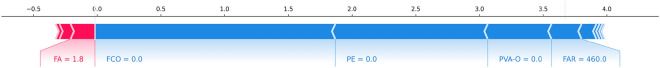
SHAP values of the individual sample.

**Figure 26:**
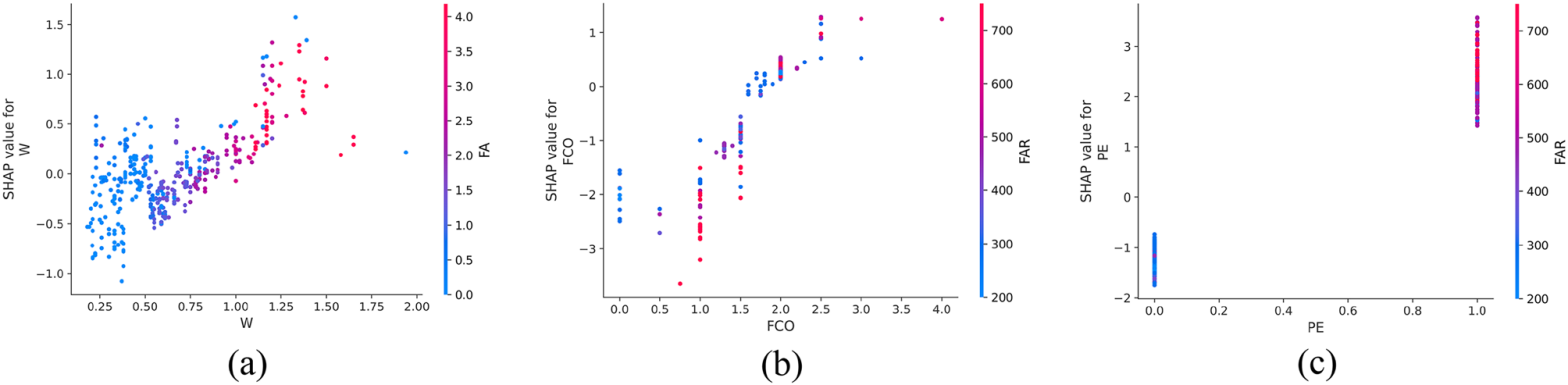
SHAP partial dependency plots for the TSC.

Figure 25 shows the SHAP values of a random single sample of the training model. The most extensive red bar was fly ash. For this specific sample, fly ash would increase the tensile strain capacity. The most extensive blue bar was the fiber content. In this particular sample, the fiber content would reduce the tensile strain capacity. Figure 26 shows the SHAP dependency partial plots. An observation can be made that the tensile strain capacity was positively correlated with the water-cement ratio, fiber content, and whether there was PE fiber. In addition, the three features were roughly linear with the tensile strain capacity, and the sensitivity between tensile strain capacity and input features was moderate and stable.

### Control strategy of ECC mechanical indicator

According to the feature importance and SHAP analysis, there are different strategies to improve each mechanical indicator of ECC.

Reducing the water-cement ratio, increasing silica fume content, and selecting PE fiber are the most effective strategies for compressive strength. For flexural strength and tensile strength, selecting PE fiber, reducing the water-cement ratio, and increasing the fiber aspect ratio are the most effective methods. For tensile strain capacity, increasing the fiber aspect ratio, selecting PE fiber, and increasing fiber content will be the most effective methods. The control methods of ECC mechanical properties are shown in Table 9. An observation can be made that there are effective methods to improve the four mechanical properties, such as the use of PE fibers and the reduction of the water-cement ratio, thereby providing effective guidance for the design of ECC.

**Table 9:** Mechanical performance control methods of ECC.

Mechanical properties	Effective method
Compressive strength	(1) Reduce the water-cement ratio (2) Increase the content of silica fume (3) Apply PE fiber
Flexural strength	(1) Apply PE fiber (2) Reduce the water-cement ratio (3) Increase fiber aspect ratio
Tensile strength	(1) Apply PE fiber (2) Reduce the water-cement ratio (3) Increase fiber aspect ratio
Tensile strain capacity	(1) Increase fiber aspect ratio (2) Apply PE fiber (3) Increase fiber content

## Conclusion

Based on the analysis using four machine-learning models, the four most critical mechanical properties of ECC were predicted, encompassing a wide range from ordinary to high compressive strength. Leveraging SHAP analysis technology, the feature importance of the optimal model was investigated and the sensitivity of feature parameters was thoroughly examined. Finally, based on the results of machine learning research, control suggestions for ECC mechanical properties were established. These findings are directly applicable to enhancing the design of ECC, facilitating its real-world application. The main conclusions are as follows:

- The RF model performed best for compressive and flexural strength prediction, followed by the XGB model. For compressive strength, the R^2^ of the RF for the training set was 0.99, and the R^2^ and MAE for the test set were 0.92 and 5.40. For the flexural strength, the R^2^ of the RF for the training set was 0.97, and the R^2^ and MAE for the test set were 0.91 and 1.62. Therefore, RF is the most recommended model for predicting ECC's compressive and flexural strength.
- In predicting tensile strength and tensile strain capacity, the XGB model performed best, followed by the random forest model. For the tensile strength, the R^2^ of the XGB model for the training set was 0.97, and the R^2^ and MAE for the test set were 0.87 and 0.68. For the tensile strain capacity, the R^2^ of the XGB model for the training set was 0.95, and the R^2^ and MAE for the test set were 0.80 and 0.84. Therefore, the XGB model is the most recommended model for predicting the tensile strength and tensile strain capacity of ECC.
- Compressive strength was positively correlated with silica fume and water reducing agent and negatively correlated with water cement ratio. PE fiber positively affected flexural strength, and the water-cement ratio had a negative effect on flexural strength. PE fiber positively affected tensile strength, while the water-cement ratio negatively affected tensile strength. PE fiber, fiber content, and oil film PVA fiber positively impacted tensile stress capacity.
- Compressive strength and tensile strength were highly sensitive to the change of W and WR, and the flexural strength was highly sensitive to the change of W. The sensitivity between tensile strain capacity and input features was moderate and stable.

From the perspective of the ECC mechanical index control strategy, the application of PE fiber is a very effective method to improve each mechanical index, and the second is to reduce the parameter of the cement ratio. In the design of ECC in practical engineering, these two characteristic parameters should be adjusted first to obtain the maximum effect with the least test. It avoids the repeated adjustment of each parameter in the mix ratio in the previous ECC design, which greatly reduces the test and time cost. At the same time, this paper only obtains the potential relationship between the characteristic parameters and the target values from many test data sets. In the future, we can consider the test data set and collect enough microstructure parameters of the fiber and matrix as the characteristic parameters to expect higher prediction accuracy.

### Supplementary Information

Supplementary Information.

## Data Availability

The datasets used and/or analyzed during the current study are available from supplementary information files.
